# Changes in the oral and nasal microbiota in pediatric obstructive sleep apnea

**DOI:** 10.1080/20002297.2023.2182571

**Published:** 2023-02-28

**Authors:** Xiaoman Zhang, Xinyi Li, Huajun Xu, Zhihui Fu, Fan Wang, Weijun Huang, Kejia Wu, Chenyang Li, Yupu Liu, Jianyin Zou, Huaming Zhu, Hongliang Yi, Su Kaiming, Meizhen Gu, Jian Guan, Shankai Yin

**Affiliations:** a Department of Otolaryngology Head and Neck Surgery & Shanghai Key Laboratory of Sleep Disordered Breathing & Otolaryngology Institute of Shanghai Jiao Tong University, Shanghai Sixth People’s Hospital Affiliated to Shanghai Jiao Tong University School of Medicine, Shanghai, China; b Department of Otorhinolaryngology-Head and Neck Surgery, Shanghai Children’s Hospital, Shanghai Jiao Tong University, Shanghai, China

**Keywords:** Pediatric obstructive sleep apnea, microbiota, upper airway, 16S ribosomal RNA, adenoid hypertrophy

## Abstract

**Background:**

Several clinical studies have demonstrated that pediatric obstructive sleep apnea (OSA) is associated with dysbiosis of airway mucosal microbiota. However, how oral and nasal microbial diversity, composition, and structure are altered in pediatric OSA has not been systemically explored.

**Methods:**

30 polysomnography-confirmed OSA patients with adenoid hypertrophy, and 30 controls who did not have adenoid hypertrophy, were enrolled. Swabs from four surface oral tissue sites (tongue base, soft palate, both palatine tonsils, and adenoid) and one nasal swab from both anterior nares were collected. The 16S ribosomal RNA (rRNA) V3–V4 region was sequenced to identify the microbial communities.

**Results:**

The beta diversity and microbial profiles were significantly different between pediatric OSA patients and controls at the five upper airway sites. The abundances of Haemophilus, Fusobacterium, and Porphyromonas were higher at adenoid and tonsils sites of pediatric patients with OSA. Functional analysis revealed that the differential pathway between the pediatric OSA patients and controls involved glycerophospholipids and amino acid metabolism.

**Conclusions:**

In this study, the oral and nasal microbiome of pediatric OSA patients exhibited certain differences in composition compared with the controls. However, the microbiota data could be useful as a reference for studies on the upper airway microbiome.

## Introduction

Obstructive sleep apnea (OSA) is a highly prevalent sleep disorder affecting about 1–4% of children worldwide and can cause cardiovascular and metabolic problems in later life [1]. Pathogenic adenoid hypertrophy and enlarged tonsils are the causes of airway obstruction, which mainly cause OSA in children.

The adenoid and tonsils, the lymphoid tissues of the upper respiratory tract, comprise the Waldeyer’s lymphatic ring. As part of the immune system, adenoid and tonsils play an important role in the immune response to infectious pathogens in the upper respiratory tract [2]. In addition, the role of microbiota in the upper respiratory tract, including oral and nasal flora, has been emphasized in the pathogenesis of OSA [3]. At present, there are few studies on the upper airway microbiome profile in pediatric OSA patients [2,4–9], and these studies mainly focus on the microbiota on the surface of adenoid, tonsils and anterior nares. It has been investigated that compared with healthy subjects, the presence of adenoid hypertrophy was related to changes in nasal microbiota composition [7]. The studies on the microbial characteristics of adenoid and tonsils focused on evaluating the paired microbial feature from tonsils and adenoid in children without infection [2,5,6], and there was no comparison between the normal population and patients with OSA. It has been confirmed that the diversity of microorganisms on the surface of adenoid was related to mucosal surface immune-related molecules and subjective discomfort symptom in children with snoring [2]. Compared with children with average SpO2 > 97%, children with average SpO2 ≤ 97% were accompanied with reduced alpha diversity and a higher abundance of *Bacteroidetes* in the surface of tonsils [8]. Therefore, it is reasonable to infer that the ecological imbalance of upper airway flora may be closely related to the presence of OSA in children. And the microbial analysis may be a promising field with great potential for diagnose OSA.

The upper airway cavity (i.e. oral and nasal cavity) is not a uniform ecosystem, instead comprising several fundamentally different niches with a high degree of microbial diversity. Although nasal cavity and nasopharynx (usually referred to as the adenoid part) are adjacent anatomical locations, the microbial communities in nasal cavity and nasopharynx are diverse and different [4,9]. Microbial communities in the oral and nasal cavity may be differentially affected by OSA via intermittent hypoxia or other mechanisms.

Considering the limited data on the diversity and abundance of the oral and nasal microbiota in pediatric OSA patients, we characterized the microbiota at four oral sites and nares sites by swab sampling, and compared them between pediatric OSA patients and controls. We aimed 1) to observe microbiota characteristics of oral and nasal activity in pediatric OSA patients and the relationship between these characteristics and anatomical structures; 2) to investigate whether there is a difference in the composition and diversity of the microbiota between pediatric OSA patients and children without obvious snoring.

## Materials and methods

### Study design and population

We performed a cross-sectional study on patients with polysomnography (PSG)-confirmed pediatric OSA and controls who did not snore and were recruited from Shanghai Sixth People’s Hospital Affiliated to Shanghai Jiao Tong University School of Medicine and Shanghai Children’s Hospital from October 2018 to January 2021. The research protocols were independently approved by the ethics committees of the two aforementioned tertiary hospitals (approval nos. 2018–073 and 2019 R061-F01, respectively). Informed consent was obtained from participants aged ≥7 years, and from the legal guardians of those aged <7 years. All subjects had no history of alcohol and tobacco use.

All pediatric OSA patients, but no controls, was diagnosed with adenoid hypertrophy by nasopharyngoscopy. According to the degree of adenoids obstruct the chonae, adenoid hypertrophy can be divided into four grades [10]. Grade 1 = adenoid obstruction of less than 25%; Grade 2 = adenoid obstruction of 25–50%; Grade 3 = adenoid obstruction of 50–75%; Grade 4 = adenoid obstructing 75% or more. All pediatric OSA patients included in the study had adenoid hypertrophy of Grade 2 or above. The inclusion criteria were as follows: aged 3–12 years; obstructive apnea hypopnea index (OAHI) > 1event/h; no obvious dietary preferences; agreed to participate in the study; and willingness of parents to complete the questionnaire.

The controls were children with congenital diseases who underwent general anesthesia in the hospital (e.g. for ear reconstruction, accessory ear, a cervical mass, or preauricular fistula without infection). Medical history, OSA-18 questionnaire and nasopharyngoscopy examination were used to ensure the controls were non-OSA. 1) The control subjects presented no clinical features of OSA (e.g. intermittent sleep breathing pauses, snoring, and/or daytime sleepiness) according to their parents; 2) The control subjects were screened by OSA-18 score. OSA-18 is an ordinal Likert scoring system with 18 items, which is used to determine the impact of OSA on children’s quality of daily life. It includes five subscales: sleep disturbance, physical symptoms, emotional distress, daytime function, and caregiver concerns, ranging from 18 (no impact on quality of life) to 126 (major negative impact) [11]. The symptom frequency is recorded on a 7-point scale: 1, absolutely none; 2, almost none; 3, rarely; 4, some; 5, often; 6, most;7, absolutely. OSA-18 score has been used in several studies on children [12–16], and it has been widely used and validated in China [17,18]. A score of > 60 indicates the presence of SDB in children [11]. A study in Taiwan has investigated that the specificity is 84% with the cut-off point of 67 [17]. In our study, we included subjects with OSA-18 score less than 60. 3) We excluded subjects with adenoid hypertrophy (an important feature of pediatric OSA) through nasopharyngoscopy examination. Other exclusion criteria included a special diet (gluten-free, casein diet, or specific carbohydrate diet); systemic disease (pulmonary, hepatic, renal, cardiovascular, gastrointestinal, or neurological disease), oral disease (dental caries or periodontal disease), or treatment for adenoid hypertrophy (tonsillectomy, adenoidectomy, corticosteroids, leukotriene antagonists); genetic/craniofacial syndromes, parents with obvious snoring or diagnosed with pediatric OSA; use of any medications, antibiotics or drugs to regulate the intestinal flora (prebiotics, synbiotics, or probiotics) during the previous four weeks, or active infections (bacteria, fungi, or viruses); and pets in the home (a known source of bacteria).

Weight and height were carefully measured using an electronic balance and plastic tape measure, while the participants were wearing light clothes and no shoes. Body mass index (BMI) was calculated as weight/height^2^ (kg/m^2^). Ultimately, 30 pediatric OSA patients and 30 normal controls were enrolled.

### Definitions of pediatric OSA

All patients with pediatric OSA were monitored by overnight standard PSG (Alice 5; Respironics, Murrysville, PA, USA) at our sleep center. In detail, during sleep from 10 pm to 6 am, electroencephalogram, electrooculogram, genioglossus electromyogram, thoracic/abdominal movement, leg movement, and percutaneous oxygen saturation (at the fingertip) were recorded by sensors. A well-trained technician manually scored the polygraphic data (OAHI, rapid eye movement, non-rapid eye movement, oxygen desaturation index (ODI), mean and minimum SpO_2_, and microarousal index(MAI)) blind to subject status according to the 2020 guidelines of Society of Otorhinolaryngology Head and Neck Surgery in Chinese Medical Association [19]. OSA severity was categorized as mild (OAHI 1–5 events/h), moderate (OAHI 5–10 events/h), or severe (OAHI≥10 events/h) [19].

### Biospecimen collection and DNA extraction

**Figure 1.**
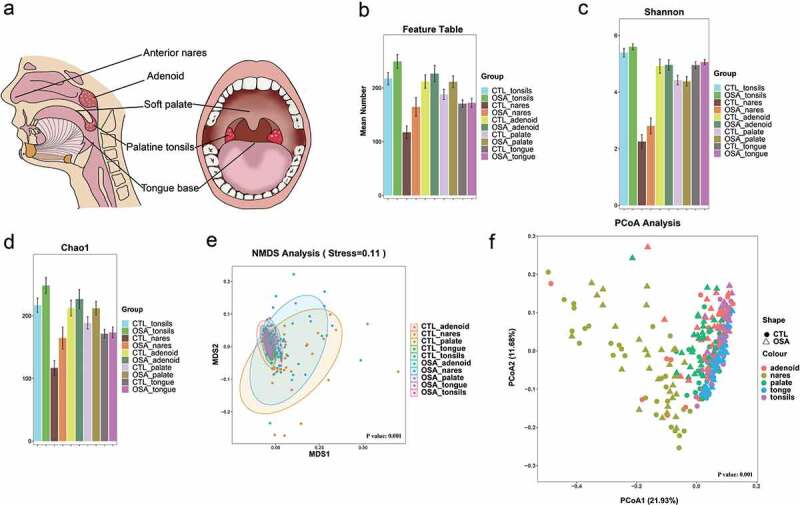
a Graphic representation of sampling from five upper airway sites in each subject. b Mean number of feature tables of pediatric OSA and controls in different upper airway sites. Group differences were assessed by using the Mann Whitney U test. *P* = 0.48 for the adenoids; *P* = 0.07 for nares; *P* = 0.25 for the palate; *P* = 0.93 for the tongue; *P* = 0.11 for the tonsils. c Shannon index between pediatric OSA and controls in different sample sites. Group differences were assessed by using the Mann Whitney U test. Shannon diversity index: *P* = 0.68 for the adenoids; *P* = 0.21 for nares; *P* = 0.72 for the palate; *P* = 0.49 for the tongue, *P* = 0.34 for the tonsils. d Chao1 index between pediatric OSA and controls in different sample sites. Group differences were assessed by using the Mann Whitney U test. Chao1 index: *P* = 0.50 for the adenoids; *P* = 0.06 for nares; *P* = 0.27 for the palate; *P* = 0.99 for the tongue, *P* = 0.12 for the tonsils. e Nonmetric multidimensional scaling analysis showed that the microbial beta diversity was significantly different. ANOSIM test was performed for comparing different groups (Stress = 0.11, overall *P* = 0.001). f Principal coordinates analysis plot based on the unweighted UniFrac distance depicting differences in the bacterial community between pediatric OSA and controls in different sample sites. ADONIS test was performed for comparing different groups (overall *P* = 0.001).

We used sterile swabs to collect four swabs from the surfaces of oral tissue sites [tongue base, soft palate, both palatine tonsils, and the adenoid (nasopharynx site)], and one nasal swab from each anterior nares (Figure 1(a)). Sterile rayon swabs were used to collect samples from participants. Participants were asked to refrain from food for 8 h and blow their nose into a tissue to clear excess secretions from the nasal cavity. These samples were taken just immediately on waking time between 6.00–-6.30am. Each swab was rotated clockwise 5 times applying constant pressure at each location for sample collection. The sample collection site was located in the ward that had been disinfected by ultraviolet. All samples were collected by the same researcher at the same locations using the same sampling technique.

The swabs were placed in a sterile container, stored on ice, and transferred to the laboratory within 1 h. The biospecimen collection procedure followed the Human Microbiome Project (HMP) (http://hmpdacc.org/doc/HMP_MOP_Version12_0_072910.pdf), as described previously [20]. Bacterial genomic DNA was extracted using a QIAamp DNA Stool Mini Kit (51504; Qiagen, Germantown, MD, USA) with same batches according to the manufacturer’s instructions. The quality and quantity of extracted DNA was detected by agarose gel electrophoresis and a NanoDrop ND-1000 spectrophotometer (Thermo Fisher Scientific, Waltham, MA, USA).

### 16S rRNA gene sequence analysis

The V3–V4 regions of the 16S ribosomal RNA (rRNA) gene were amplified from the microbial genomic DNA by polymerase chain reaction (PCR). The barcoded forward primer was 341F (5’-CCTACGGGNGGCWGCAG-3’) and the reverse primer was 805 R (5’- GACTACHVGGGTATCTAATCC-3’). Template DNA (50ng), primer mix (5 μL), and Phusion Hot start flex 2× master mix were mixed for the polymerase chain reaction (PCR; total reaction volume, 25 μL). PCR was performed using the following cycle conditions: an initial denaturation step at 98°C for 30 s followed by 35 cycles of denaturation at 98°C for 10 s, annealing at 54°C for 20 s, and elongation at 72°C for 45 s and then a final elongation step at 72°C for 10 min. Three PCRs were performed for all samples, and all PCR products were quantified by Qubit (Invitrogen, USA). The amplicons were purified using AMPure XT beads (Beckman Coulter Genomics, Danvers, MA, USA) and 0.8% agarose gels. PCR amplicons were sequenced to generate 250 bp paired-end reads using the Illumina NovaSeq platform according to the manufacturer’s recommendations (LC-Bio Technology Co., Ltd., Hangzhou, China).

### Bioinformatics and statistical analyses

Analysis of the oral and nasal microbiota was performed as described previously [21]. The raw 16S rRNA gene amplicon sequences were processed and analyzed using Quantitative Insights Into Microbial Ecology (QIIME) platform [22]. The operational taxonomic units (OTUs) (≥97% identity) were created in the Quantitative Insights into Microbial Ecology (QIIME; http://qiime.org/scripts/pick_otus.html) database. The taxonomy of OTUs was annotated according to the Greengenes database [23]. First, the sequences were demultiplexed based on the barcodes assigned to each sample. Then, the demultiplexed pair-end sequences from each sample were quality-controlled (stitched, filtered, trimmed, and de-noised, with ambiguous/chimeric sequences being removed) using DADA2 in QIIME2 and clustered to generate an amplicon sequence variant table [24].

Alpha diversity provides overall characteristics of the microbial community in individual samples and represents the richness and evenness of the sample, and beta diversity is an indicator to compare the similarity of microbial composition among samples [25]. Alpha diversity analysis of all samples was carried out using the Chao1 and Shannon diversity indices to calculate the richness and evenness index of microbial flora. Beta diversity was investigated through nonmetric multidimensional scaling (NMDS) analysis according to Bray-Curtis distance matrices. The bacterial composition (unweighted UniFrac distance) in the microbiome community was examined using the Mantel test. The analysis of similarities (ANOSIM) method and permutational multivariate analysis of variance (PERMANOVA/ADONIS) were used to predict the differences in microbiome community based on groups and sites. Differences in the relative abundance of taxa were identified based on the linear discriminant analysis effect size (LEfSe) [26]. Regarding potential functional implications, Phylogenetic Investigation of Communities by Reconstruction of Unobserved States 2 (PICRUSt2) was used to predict microbial metabolic pathways [27].

It was estimated that 20 subjects per group would be necessary to detect differences in unweighted pairwise distances with 90% power [28]. Therefore, our study (30 subjects/group) had sufficient power to detect differences in taxa between pediatric OSA patients and control subjects.

Statistical analyses were performed using the SPSS (version 22.0; IBM Corp., Armonk, NY, USA) and R studio (R Foundation for Statistical Computing, Vienna, Austria) software packages. Categorical variables are presented as numbers and percentages, and continuous variables as medians with interquartile range. The pediatric OSA and controls groups were compared using the Mann-Whitney *U* test, t test or chi-square test, as appropriate. A two-sided P value <0.05 was considered significant.

## Results

### Patient demographics

In total, 30 pediatric OSA patients and 30 control subjects were included in our study. No significant differences in age, gender, or BMI were observed between the two groups (all *P* > 0.05, Table 1). The detailed clinical and PSG data of the pediatric OSA patients are presented in Table 1. In total, 298 swab samples (60 from the tongue base, 60 from the soft palate, 60 from the palatine tonsils, 58 from the adenoids, and 60 from the anterior nares) were eligible for 16s RNA analysis.

**Table 1.** Basic characteristics of the overall population.

Variable	Case (n=30)	Control (n=30)	P value
**Demographics**			
Age, years	7(5–8)	6(3–10)	0.395
Male (%)	16(53.3%)	16(53.3%)	0.999
Height, m	1.27(1.2–1.4)	1.18(1.1–1.4)	0.544
Weight, Kg	22.8(18.4–31.3)	22.6(17.8–36.1)	0.923
BMI, Kg/m**2**	15.2(14.0–17.0)	16.6(14.7–17.9)	0.102
**Sleep apnea**			
OAHI	5.8(3–10.1)	-	-
OAHI_REM_	7.3(3.4–15.9)	-	-
OAHIN_REM_	4.1(2.4–10.1)	-	-
Average SaO_**2**_	97(94.8–98)	-	-
Minimum SaO_**2**_	87.5(84–91)	-	-
ODI	3.0(1.9–5.8)	-	-
MAI	11.8(7.9–28.6)	-	-

The data are presented as means and standard deviation; skewed data are presented as the median (IQR), and categorical data as the number (percentage). Differences in the baseline characteristics among the two groups were examined using Mann-Whitney *U* or Chi-square test as appropriate.

**Abbreviations**: BMI, body mass index; OAHI, obstructive apnea-hypopnea index; OAHI_REM_, rapid eye movement obstructive apnea-hypopnea index; OAHI_NREM_, non-rapid eye movement obstructive apnea-hypopnea index; SaO2, oxygen saturation; ODI, oxygen desaturation index; MAI, micro-arousal index.

### Differential upper airway microbiota diversity was observed between the pediatric OSA patients and controls

A total of 24,912,788 raw sequences were acquired. After sequence processing, 21100,111 high-quality 16S rRNA gene sequences (accounting for 84.7% of the raw sequences) were identified, with a median read count of 71,381 (range: 48649–84,926) per sample. A total of 57,438 feature tables were generated from these samples (13–437 tables per sample). The Mann Whitney U test detected no differences in feature tables between the pediatric OSA and control groups at the various upper airway sites (*P* = 0.48 for the adenoids; *P* = 0.07 for nares; *P* = 0.25 for the palate; *P* = 0.93 for the tongue; *P* = 0.11 for the tonsils) (Figure 1(b)).

The Chao1 and Shannon diversity indices were not significantly different between the pediatric OSA and control groups at the various upper airway sites (Chao1 index: *P* = 0.50 for the adenoid; *P* = 0.06 for nares; *P* = 0.27 for the palate; *P* = 0.99 for the tongue, *P* = 0.12 for the tonsils; Shannon diversity index: *P* = 0.68 for the adenoid; *P* = 0.21 for nares; *P* = 0.72 for the palate; *P* = 0.49 for the tongue, *P* = 0.34 for the tonsils) (Figure 1(c,d)). Overall, the microbiota alpha-diversity values were similar, indicating comparable alpha diversity of the microbiomes at these upper airway sites between the pediatric OSA and control groups.

Microbiota beta-diversity was compared between the pediatric OSA and control groups at the various upper airway sites by NMDS analysis and principal coordinate analysis (PCoA). (Figure 1(e,f)). The dimension reduction analysis indicated significant dissimilarity in the microbial communities of tongue base, soft palate, tonsils, and adenoid (*P* = 0.006 for the adenoid; *P* = 0.001 for the palate; *P* = 0.008 for the tongue, *P* = 0.001 for the tonsils) (Figure S1). In addition, the pathogenic bacteria from the five upper airway sites were different in control groups and in pediatric OSA group (*P* = 0.001 for controls; *P* = 0.001 for pediatric OSA) (Figure S1).

### Bacterial composition identified from the different upper airway sites between pediatric OSA patients and controls

**Figure 2.**
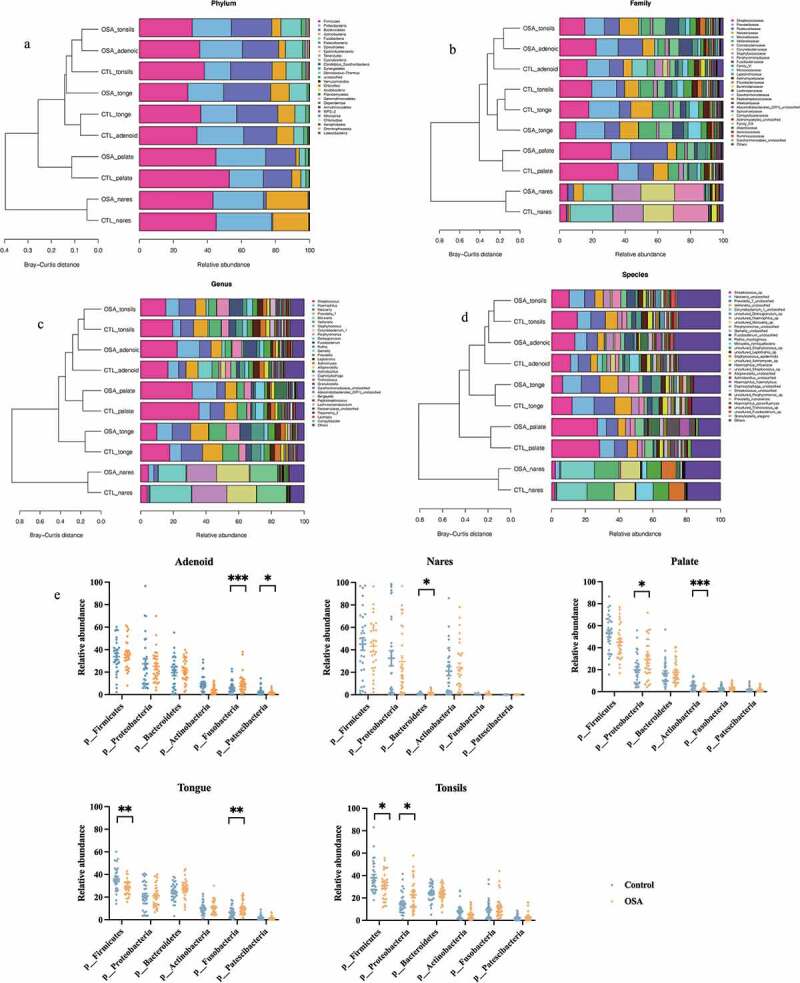
Average composition of upper airway bacterial composition at the phylum, family, genus, and species level between pediatric OSA and controls in different sample sites. a at the phylum level, b at the family level, c at the genus level, d at the species level. e analysis of phylum abundance of six predominant bacteria. Data were expressed as mean ± SEM. Group differences were assessed by using the t test. * *P* < 0.05, ** *P* < 0.01, *** *P* < 0.001.

In Figure 2(a-d), the average composition of bacterial communities at the phylum, family, genus, and species levels were presented. In order to evaluate the difference between the groups more intuitively, we next introduced the analysis of phylum abundance of predominant bacteria (Figure 2(e)). The results have shown that the relative abundance of *Fusobacteria* and *Patescibacteria* were different in the adenoid site between control subjects and pediatric OSA patients, while *Bacteroidetes* in nares sites, *Proteobacteria* and *Actinobacteria* in the palate site, *Firmicutes* and *Fusobacteria* in the tongue site, and *Firmicutes* and *Proteobacteria* in tonsils sites were significantly different between control subjects and pediatric OSA patients.

### Differences in taxa between the pediatric OSA and control groups at the various upper airway sites

**Figure 3.**
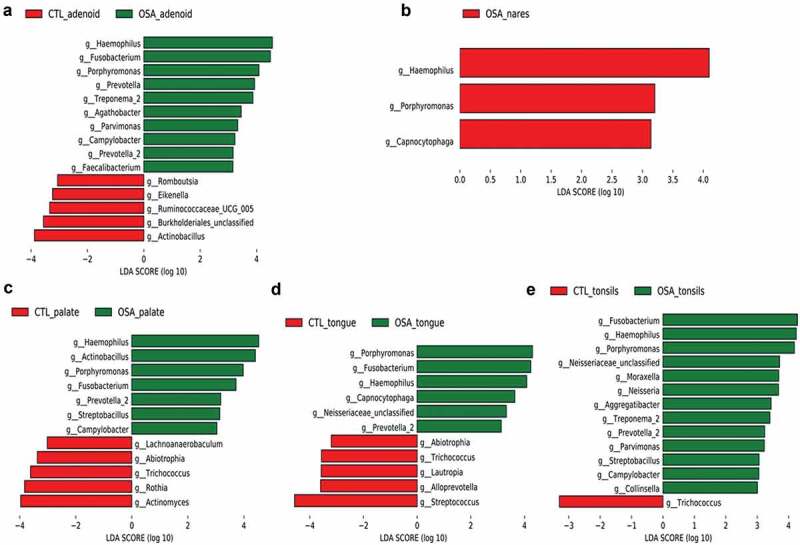
Differentially abundant bacterial taxa identified by linear discriminant analysis (LDA) coupled with effect size measurements (LEfSe) in comparisons of pediatric OSA and controls. a in adenoid site. b in nares site. c in palate site. d in tongue site. e in tonsils site. LEfSe scores measure the consistency of differences in relative abundance between taxa in the groups analyzed (control vs OSA), with a higher score indicating higher consistency. LDA score>3 and P < 0.05 were considered to be significant. The bacterial taxa shown in the figure were all significantly different between the controls and pediatric OSA.

Linear discriminant analysis effect size (LEfSe), which is a method for discovering biomarker, was used to identify genera that best characterize each group [29]. We utilized LEfSe analysis to compare the microbiota between the pediatric OSA and control groups at the various upper airway sites (Figure 3(a-e)). We identified important taxonomic differences between the pediatric OSA and control groups based on a logarithmic linear discriminant analysis score (log10) > 3.0. The LEfSe results suggested remarkable differences in upper airway microbiota between the pediatric OSA and control groups. We were particularly interested in differences in taxa at the genus level. In the adenoids, the relative abundances of the genera *Haemophilus, Fusobacterium, Porphyromonas, Prevotella, Treponema, Agathobacter, Parvimonas, Campylobacter, and Faecalibacterium* were higher in the pediatric OSA than control group, whereas the relative abundances of genera *Actinobacillus, Burkholderiales, Ruminococcaceae_UCG_005, Eikenella*, and *Romboutsia* were higher in the controls than pediatric OSA (Figure 3(a)). In nares, only the relative abundances of the genera *Haemophilus, Porphyromonas*, and *Capnocytophaga* were higher in the pediatric OSA than control group (Figure 3(b)). In palate, the relative abundances of the genera *Haemophilus, Actinobacillus, Porphyromonas, Fusobacterium, Prevotella, Streptobacillus, and Campylobacter* were higher in the pediatric OSA than control group, whereas the relative abundances of genera *Lachnoanaerobaculum, Abiotrophia, Trichococcus, Rothia, and Actinomyces* were higher in the controls than pediatric OSA (Figure 3(c)). In tongue, the relative abundances of the genera *Porphyromonas, Fusobacterium, Haemophilus, Capnocytophaga and Prevotella* were higher in the pediatric OSA than control group, whereas the relative abundances of genera *Abiotrophia, Trichococcus, Lautropia, Alloprevotella, and Streptococcus* were higher in the controls than pediatric OSA (Figure 3(d)). In tonsils, the relative abundances of the genera *Fusobacterium, Haemophilus, Porphyromonas, Moraxella, Neisseria, Aggregatibacter, Treponema, Prevotella, Parvimonas, Streptobacillus, Campylobacter*, and *Collinsella* were higher in the pediatric OSA than control group, whereas only the relative abundance of genera *Trichococcus* was higher in the controls than pediatric OSA (Figure 3(e)).

### Functional prediction of microbiota at the different upper airway sites in the pediatric OSA and control groups

**Figure 4.**
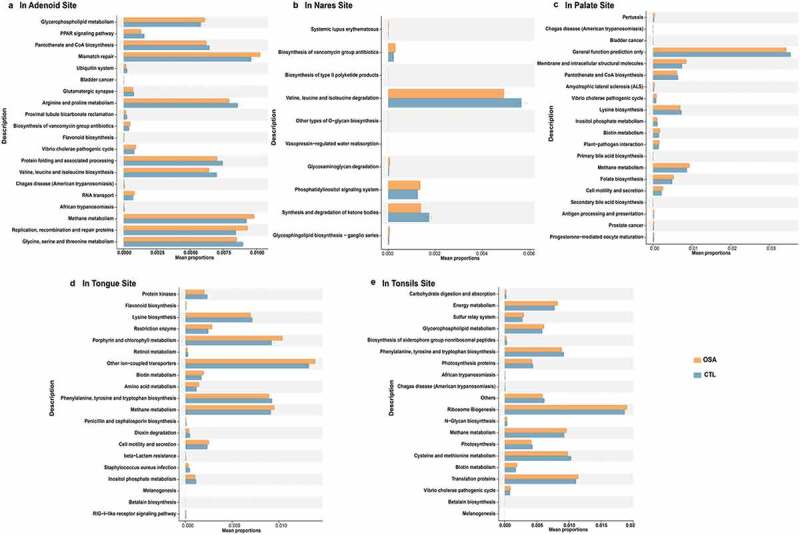
The bacterial functions of pediatric OSA and controls in different sample sites were predicted by PICRUSt2 analysis. a in adenoid site. b in nares site. c in palate site. d in tongue site. e in tonsils site. The pathways shown in the figure were all significantly different between the controls and pediatric OSA (P < 0.05).

PICRUSt analyses were performed to explore functional and metabolic variations in the upper airway microbial communities, and the results are presented in Figure 4(a-e). The PICRUSt analysis identified 20 Kyoto Encyclopedia of Genes and Genomes (KEGG) pathways with significant differential abundance between pediatric OSA and controls in adenoid (Figure 4(a)), 10 KEGG categories in nares (Figure 4(b)), 20 KEGG categories in palate (Figure 4(c)), 20 KEGG categories in tongue (Figure 4(d)), and 20 KEGG categories in tonsils (Figure 4(e)), respectively. Compared with the control group, the function of adenoid flora in pediatric OSA patients was concentrated in glycerophospholipid metabolism and amino acid metabolism, including arginine and proline metabolism, valine, leucine and isoleucine biosynthesis, and glycine, serine and threonine metabolism (Figure 4(a)). Compared with normal controls, the function of tonsils flora in pediatric OSA patients was also characterized by the disturbance in glycerophospholipid metabolism and decline of amino acid metabolism (Figure 4(e)). In addition, the flora function of nares, tongue, palate, and tongue in pediatric OSA patients was disturbed in amino acid metabolism (Figure 4(b-d)). Similar patterns of differentially enriched genes functioning in amino acid biosynthesis, metabolism, and signal transduction were detected at the upper airway sites in both groups. The enriched pathways were related to human pathogenesis, as expected.

## Discussion

This is the first study to provide an overview of the differences in upper airway microbiota between pediatric OSA patients and controls. We observed certain microbial differences and bacterial functions between pediatric OSA patients and controls at the various upper airway sites using microbiome 16S rRNA gene sequencing. A few studies have used the method of bacterial culture to determine the most prevalent species [30,31]. However, strict growth conditions are needed for certain bacterial culture, and cannot be simulated in the laboratory, so microbiota in vitro cannot always be detected by culture-dependent methods [32].

16S rDNA refers to the DNA sequence coding the ribosomal 30S subunit. The gene has a total length of about 1542bp and is composed of 9 variable regions and 10 conserved regions (the variable regions are V1 to V9) [33]. The sequence of conserved regions is highly conserved, while the variable region sequence varies from species to species [33]. Moreover, the degree of variation is closely related to the bacterial taxonomy. Therefore, it is considered to be one of the indicators for bacterial classification and identification.

In this study, we used the second-generation sequencing sequences to amplify V3 and V4 regions of 16S rRNA gene, only about 400 bases. Due to the lack of reference sequences, most of the sequences could only be classified to the taxonomic level of ‘Genus’ or above, and the diversity and composition ratio of microbiota at the level of ‘Genus’ or above could be obtained. In addition, the sequences of 16S rDNA genes are similar between species, so it is impossible to accurately identify species only by part of 16S rRNA sequencing. Therefore, even though the data from 16S rRNA sequencing has shown the certain difference of the oral and nasopharyngeal microbiota in pediatric OSA patients, we speculated that the specific characteristics of the microbiota in pediatric OSA patients might be masked due to technical reasons. It might be that one or several microflora species, especially from the adenoid and tonsil sites, did play a critical role in promoting proliferation and hypertrophy, but these features were not presented for technical reasons.

Many studies have focused on the effects of environmental factors on the oral and nasal microbiota, such as cigarette smoking and alcohol consumption [20,34,35]. Exposure to these factors could lead to a higher abundance of opportunistic pathogens in the upper respiratory tract [20,34,35]. Sleep can also affect microbial abundance in the pediatric oral cavity [36]. OSA is a common chronic sleep disorder characterized by intermittent hypoxia during sleep. The abundance of oral microbiota changes during the anoxia and reoxygenation cycle. Microbiome profiling has played an integral role in understanding the development and exacerbation of OSA [3]. In our previous study, we reported that the microbiota (*Firmicutes, Proteobacteria, Bacteroidetes, Fusobacteria*, and *Actinobacteria*) on the buccal mucosa in pediatric OSA patients was altered [37]. However, in this study, the relationship between the microbial characteristics of different parts and the presence of OSA was minor. There were two reasons: First, the presence of OSA might affect different parts. For example, the presence of OSA might only affect the microbial characteristics of other parts of the sample, while the surface flora of these five parts sampled in this study only changed slightly. This is similar to cigarette smoking, which only affects the oral microbiota in the buccal mucosa [20]. Besides, it might be that one or several microflora species, especially from the adenoid and tonsil sites, did play a critical role in promoting proliferation and hypertrophy, but these features were not presented for technical reasons. Finally, one study showed that the salivary microbial composition was stable after antibiotic use at various follow-up timepoints [38]. Thus, oral microbial organisms may be tolerant to many adverse conditions [39]. This could partially explain why the microbial communities in the upper airway are resistant or resilient to disturbances such as intermittent hypoxia.

Data on the effect of OSA on nasal or nasopharynx microbiota are sparse, and the results are inconsistent. One study found that the nasal microbiome of adult patients with severe OSA was enriched with *Streptococcus, Prevotella*, and *Veillonella* [40]. Another study reported no difference in the composition of the nasopharynx microbiome between mild-to-moderate adult OSA patients and those without OSA [41]. Only one study has explored the altered composition of the nasal microbiome in pediatric OSA patients [7], while one study has found no significant difference in nasopharynx or nasal cavity composition or diversity between patients with chronic rhinosinusitis and controls [9].

The adenoids and tonsils are lymphoid tissues; hypertrophy thereof plays an important role in the onset and development of pediatric OSA, but few studies have explored the adenoid or tonsil microbiome in OSA to determine whether it participates in hypertrophy. Because pediatric OSA patients concerned in our study were all accompanied by adenoid hypertrophy, we mainly focused on the flora characteristics at adenoids and tonsils sites. Higher abundances of the genera *Haemophilus, Fusobacterium*, and *Porphyromonas* were found in the adenoids and tonsils of our pediatric OSA group.

*Fusobacterium* is one of the symbiotic bacteria mainly colonized the oral and colonic mucosa of human and animal [42]. *Fusobacterium* is greatly associated with anaerobe infections of head and neck in children [43]. In addition, *Fusobacterium nucleatum* infection is prevalent in colorectal carcinoma [44]. *Fusobacteria* has been detected at a tonsillar site in pediatric OSA patients and associated with the AHI [40,45]. *Fusobacterium nucleatum* could promote tumor cell proliferation through various mechanisms: 1) The combination of FadA and E-cadherin derived β-Catenin/Wnt activation [46]; 2) The activation of the LPS-TLR4-NF-κB pathway promoted the expression of miR21, which directly regulated the expression of anti-oncogenes [46]; 3) The outer membrane vesicles activated TLR4 to promote the expression of inflammatory factors [47]. Therefore, it was reasonable to suspect that the increase of *Fusobacterium* in the oral and nasal cavity of pediatric OSA might be closely related to the hypertrophy of adenoids. However, the causal relationship between them was still unclear, that was, whether the change of the abundance of *Fusobacterium* leads to the hypertrophy of adenoids, or whether the hypertrophy of adenoids leads to the change of the oral and nasal cavity environment, thereby causing the change of the abundance of *Fusobacterium.*

*Haemophilus* mainly inhabits the pharynx and oral mucosa of human and animals, and can cause primary suppurative infection and serious secondary infection [48]. *Haemophilus influenzae* is one of the most common causes of acute otitis media [49]. *Haemophilus* is the main pathogenic biofilm bacteria constituting the adenoid reservoir [50,51]. *Haemophilus influenzae* is a pathogen that mainly affects children. It can cause serious infections, including sepsis and bacterial pneumonia [52]. The abundance of *Haemophilus i*nfluenzae increased in the nasopharynx of children with increased risk of wheeze [53]. Therefore, *Haemophilus influenzae* might be closely related to abnormal immunity of upper airway mucosa in children.

The abundance of *Porphyromonas* is higher in patients with OSA. It profoundly affects the likelihood of developing OSA-related cardiovascular diseases [54]. It has been reported that the role of *Porphyromonas gingivalis* was mainly related to oxidative stress [55]. In dental plaque, *Porphyromonas gingivalis* could stimulate macrophages to produce the expression of TNF α, IL-6 and nitric oxide [55]. In vascular endothelial cells, *Porphyromonas gingivalis* could also promote the expression of IL-1β, IL-6, and TNF α [56]. The increase of proinflammatory factors might be one of the causes of adenoid hypertrophy. The roles of these genera in the development of the adenoids and tonsils need to be further explored.

After the analysis of species composition, alpha diversity, and beta diversity, it is essential to analyze the different functions between two groups. Functional analysis is more important than separate species composition analysis in terms of biological significances, and can provide more reference information for downstream mechanism. PICRUSt is an algorithm that uses 16S spectrum to predict the functional composition of microbial community metagenome [57]. PICRUSt2, developed on the basis of PICRUSt1, includes the steps of optimizing genome prediction, putting sequences into reference phylogeny instead of limited to reference OTUs [27]. Therefore, PICRUSt2 can provide higher accuracy and flexibility for marker gene metagenomes.

In view of the fact that pediatric OSA patients have been diagnosed with adenoid hypertrophy in this study, the prediction of the bacterial functions at adenoids sites can directly reveal the pathological mechanism of adenoid hypertrophy. Compared with the control group, the function of adenoid flora in pediatric OSA patients was concentrated in glycerophospholipid metabolism (Figure 4(a)). Glycerophospholipids are the most abundant phospholipids in the body [58]. In addition to forming biofilms, glycerophospholipids are also one of the components of bile and membrane surfactant, and participate in the recognition and signal transduction of proteins by cell membranes [58,59]. Glycerophospholipids can regulate Immune on bacterial LPS through TLR4 [60,61]. Several studies have shown that glycerophospholipid metabolism played a role in LPS-induced sepsis and subsequent organ damage [60,62]. The disturbance of glycerophospholipid metabolism may be related to the inflammatory response caused by dysbiosis of the flora. The disturbance of glycerophospholipid metabolism pathway could also be seen in the prediction function of tonsil flora (Figure 4(e)). These results suggested that dysbiosis might play a role in the development of adenoid hypertrophy in children.

Compared with normal controls, the function of adenoid flora in pediatric OSA patients was also characterized by the decline of amino acid metabolism, including arginine and proline metabolism, valine, leucine and isoleucine biosynthesis, and glycine, serine and threonine metabolism. In addition, the flora function of nares, tongue, palate, tongue, and tonsils in pediatric OSA patients was disturbed in amino acid metabolism, which was consistent with our previous research results on oral flora in pediatric OSA patients [37]. Our previous review also found that amino acid metabolism in pediatric OSA patients is more common than in adult patients [3].

In addition, Spearman correlation was performed between the abundance of microbiota at different sites and PSG-derived sleep variables of pediatric OSA patients (Figure S2), and the results reflected that the microbiota characteristics related to sleep variables at different sites had overlap to some extent except for those at the nares. The direct communication between the nares and the environment outside might be one of the main reasons. The microbiota features related to OSA at other sites, especially the adenoids, tonsils and palates, may provide new sights for the pathogenesis and diagnostic biomarkers of pediatric OSA.

*Veillonella*, gram-negative anaerobic genus, mainly distributes in the oral cavity, pharynx, respiratory tract and digestive tract [42]. It was identified as one of the predominant genera in dental plaque of preschool children [63]. It has been confirmed that *Veillonella* might play an important role in infection and immune development. The expression of TLR-2, TLR-4 and IL-1β in the mucosa were directly related to *Veillonella spp*. counts [64]. A study of nasopharyngeal swabs from children diagnosed with acute respiratory disease revealed that the presence of *Veillonella parvula* was associated with pneumonia [65]. In addition, *Veillonella parvula* could cause macrophage-related inflammation through LPS-TLR4 pathway [66]. Therefore, it was reasonable to suspect that *Veillonella* might play a key role in the development of the immune system in early childhood. In our study, the genus *Veillonella* at the adenoids, palates and tongues sites was related to AHI, ODI and mean SaO2. It was not difficult to understand that intermittent hypoxia caused by upper respiratory tract obstruction caused a decrease in SaO2, thus promoting the propagation of this anaerobic genus. However, whether *Veillonella* genus could promote adenoid hypertrophy through LPS-TLR4 pathway might require further exploration in subsequent studies.

*Atopobium* is featured by anaerobe, and few studies has investigated the effects of *Atopobium* on the upper respiratory tract diseases. *Atopobium* has been proven to be associated with periodontitis [67,68]. However, there is still controversy indicating no significant association between *Atopobium* and periodontitis [69]. In addition, studies have shown that *Atopobium* is enriched in oral samples from patients with esophageal squamous cell carcinoma and pancreatic cancer [70,71]. In a prospective study of non-smoking women, oral *Atopobium* was positively correlated with cancer risk [72]. Compared with healthy subjects, the genus *Atopobium* was increased in oral tissues of patients with oral squamous cell carcinoma (OSCC), and functional prediction analysis revealed enrichment of pro-inflammatory bacterial attributes including LPS biosynthetic pathway [73]. In our study, *Atopobium* at the adenoid site was observed to be negatively correlated with the mean SaO2, which corresponded to its anaerobic attribute. Due to the lack of research on *Atopobium*, the relationship between ectopic colonization of *Atopobium* and adenoid hypertrophy is not clear.

*Granulicatella*, *Alloprevotella*, *Tannerell* and *Filifactor* were considered to be periodontal disease related genera [67,68,74,75]. *Granulicatella*, classified as facultative anaerobic genus, is detected in many sites of the oral cavity [76]. *Granulicatella* was confirmed to be closely related to infection. In addition to oral diseases, it was also involved in a variety of infections, including infective endocarditis and maxillary sinusitis [77,78]. Several studies have reflected that *Alloprevotella sp*. is enriched in patients with oral cancer [79,80]. *Filifactor alocis* was related to OSCC and the abundance of this species increased with the progress of OSCC [81]. These results indicated that these genera might actually participate in oral carcinogenesis and development. *Tannerella forsythia* was thought to induce increased expression of glucose transporters 1 and glucose transporters 4 in cancer cells, thereby increasing nutrient supply and promoting proliferation [82]. In addition, *T. Forsythia* has been proven to induce the production of the proinflammatory cytokine IL-8 [83]. In this study, *Granulicatella*, *Alloprevotella*, *Tannerell* and *Filifactor* were considered to be related to sleep variables. The possible explanation was that *Granulicatella*, *Alloprevotella*, *Tannerell* and *Filifactor* ectopically colonized at the adenoids and tonsils sites, which led to the adenoids and tonsils hypertrophy through their pro-inflammatory and pro-proliferation effects, and then caused obstruction of the upper respiratory tract.

This study innovatively explored the genus bacteria significantly related to sleep variables in the oral cavity of pediatric OSA patients. Most of these genera are periodontal disease-related pathogens, and exist in dental plaque. In addition, these genera are closely related to the pro-inflammatory and pro-proliferation effects. However, in our study, these genera have migrated to the surface of the palates, adenoids and tonsils in pediatric OSA patients, and the roles of these genera have not yet been determined. Therefore, it is uncertain whether the ectopic colonization of these genera is caused by the decrease of SaO2 induced by OSA or the ectopic pathogen causes the inflammatory infiltration of adenoids and tonsils, further leading to hyperplasia and hypertrophy. In conclusion, although 16S rRNA sequencing has shed light on the pathogenesis of adenoid hypertrophy, future studies still need to further verify the specific effects of these genera through pathogenic experiments and molecular experiments.

Several limitations of this study should be acknowledged. First, we used 16S rRNA sequencing (rather than deep-shotgun sequencing) to detect microbial diversity, which is not capable of in-depth analysis of the function of the upper airway microbiome. The mutual validation between metagenome sequence and bacterial culture may provide more solid evidence for future research. Second, although the sample size was sufficient to detect differences in microbes between the case and control groups, our cohort was relatively small. Thus, caution is required when interpreting the data. Third, the normal controls were classified as such based on OSA-18 score rather than objective sleep parameters (i.e. standard PSG). Fourth, various confounding factors, such as food intake, eating habits, and mouth breathing during sleep were not analyzed. Finally, our study used a case-control design, so causality could not be inferred. The role of the upper airway microbiota in pediatric OSA merits further exploration. Despite these limitations, our results constitute useful information for future studies exploring the role of upper airway floral disturbances in pediatric OSA.

## Conclusions

This study revealed certain differences in the upper airway microbiota between pediatric OSA patients and controls. Large-scale metagenomic studies are warranted for in-depth examination of the upper airway microbiota in pediatric OSA patients.

## Supplementary Material

Supplemental Material

## Data Availability

The datasets used and/or analyzed during the current study are available from the corresponding author on reasonable request.
