# Supplementary material for: Changes in the oral and nasal microbiota in pediatric obstructive sleep apnea
Source: J Oral Microbiol. 2023 Feb 28;15(1):2182571. doi: 10.1080/20002297.2023.2182571 (PMC9980019; doi:10.1080/20002297.2023.2182571)
Supplement: Supplemental Material [file ZJOM_A_2182571_SM2716.zip › Supplementary files/Supplementary materials.pdf]

Supplementary materials

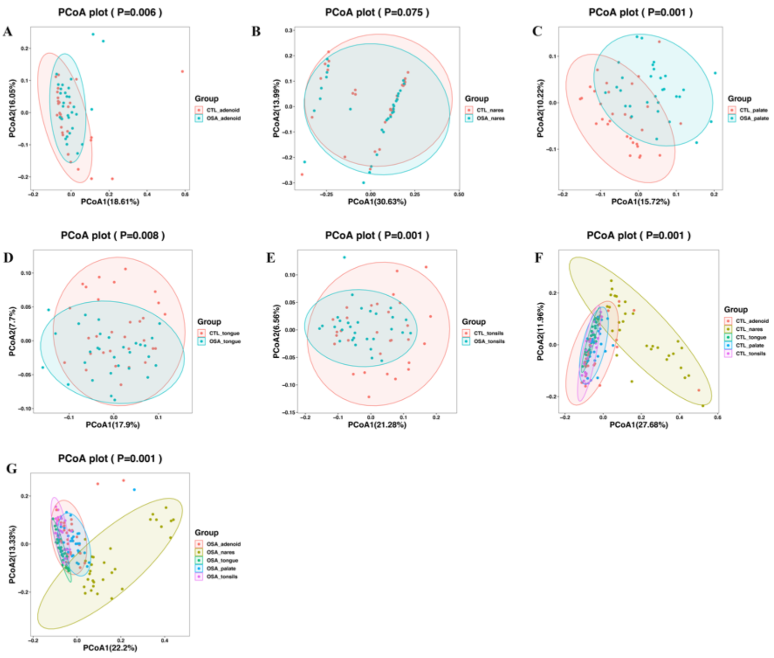

Figure S1. Principal coordinates analysis (PCoA) plot based on the unweighted UniFrac distance depicting differences in the bacterial community between pediatric OSA and controls in different sample sites. ADONIS test was performed for comparing different groups. **a** in adenoid site. **b** in nares site. **c** in palate site. **d** in tongue site. **e** in tonsils site. **f** five sites in oral and nasal cavity in control group. **g** five sites in oral and nasal cavity in pediatric OSA group.

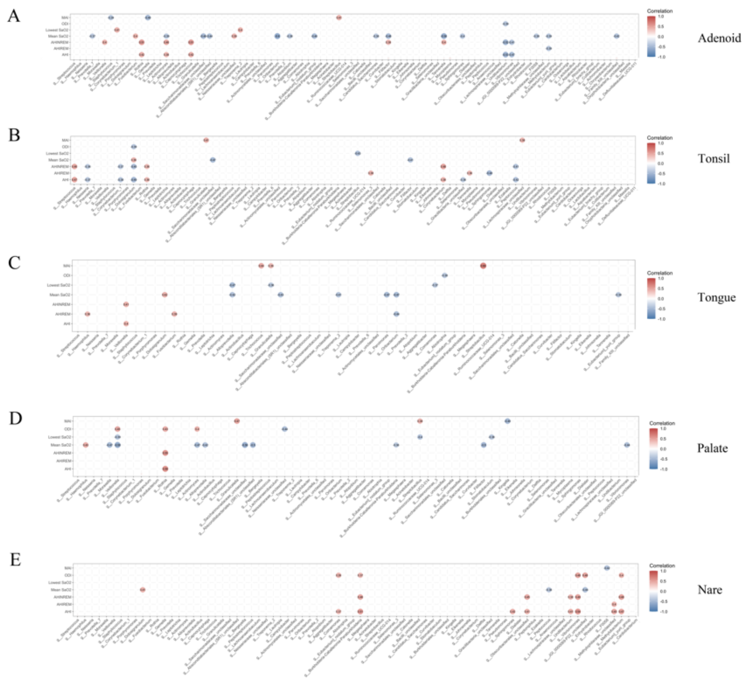

Figure S2. Genera significantly correlated with sleep variables in pediatric OSA patients by Spearman correlation. **a** in adenoid site. **b** in tonsils site. **c** in tongue site. **d** in palate site. **e** in nares site. P<0.05, and numbers represented correlation coefficient. AHI, obstructive apnoea hypopnea index; ODI, oxygen desaturation index; MAI, micro-arousal index.
