# Supplementary figures and images for: Changes in the oral and nasal microbiota in pediatric obstructive sleep apnea
Source: J Oral Microbiol. 2023 Feb 28;15(1):2182571. doi: 10.1080/20002297.2023.2182571 (PMC9980019; doi:10.1080/20002297.2023.2182571)

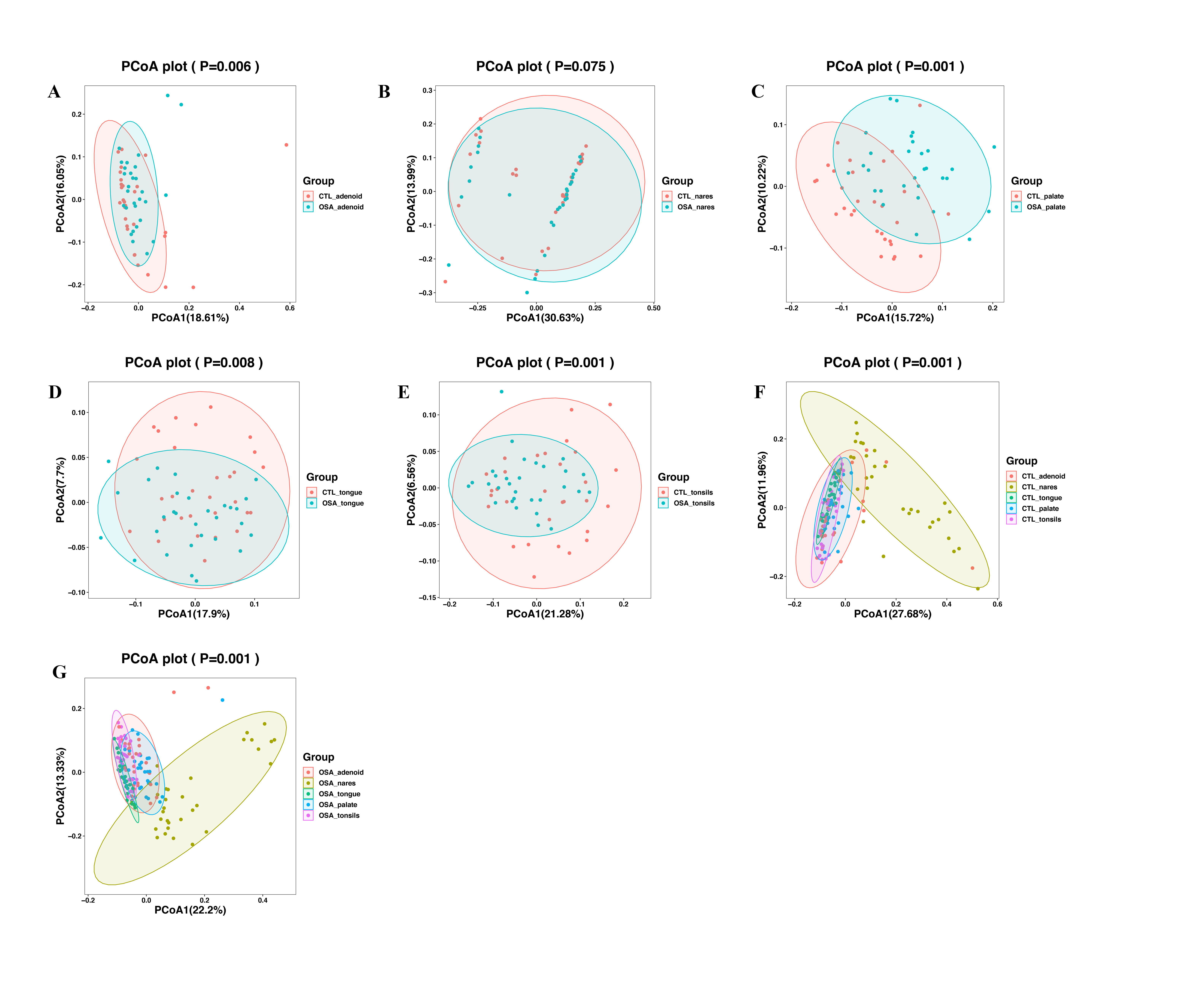

Supplement: Supplemental Material [file ZJOM_A_2182571_SM2716.zip › Supplementary files/figure S1.tif]

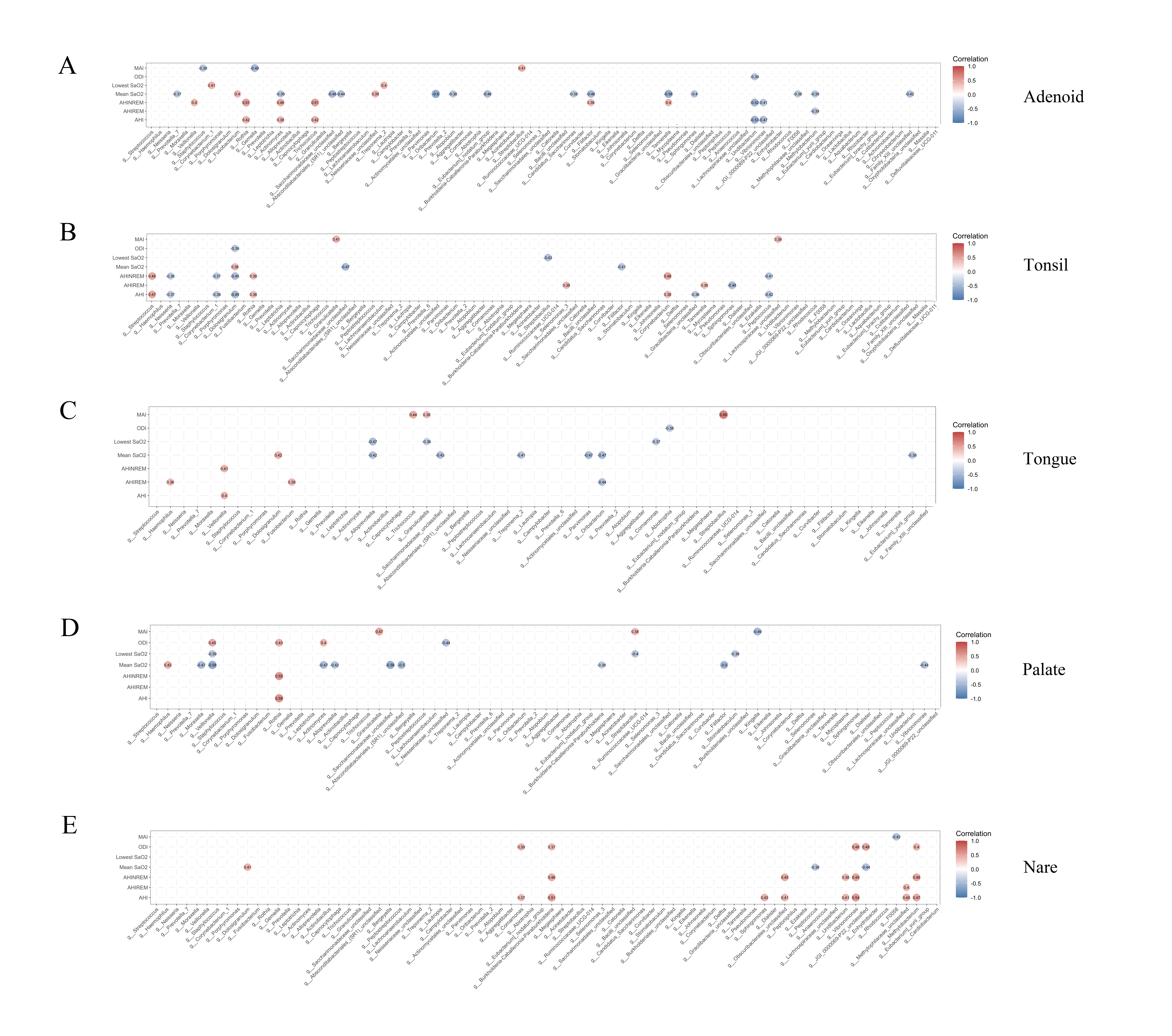

Supplement: Supplemental Material [file ZJOM_A_2182571_SM2716.zip › Supplementary files/Figure S2.tif]
